# Supplementary material for: EEG evidence that morally relevant autobiographical memories can be suppressed
Source: Cogn Affect Behav Neurosci. 2022 Aug 19;22(6):1290–310. doi: 10.3758/s13415-022-01029-5 (PMC9622558; doi:10.3758/s13415-022-01029-5)
Supplement: Supplementary file 1 — (DOCX 223 kb) [file 13415_2022_1029_MOESM1_ESM.docx]

**Supplementary Methods**

**Session 2 – Tasks conducted before and after the critical Think/No-Think phase**

**Practice Think/No-Think phases.** The aim of this phase was to allow participants to practice the Think/No-Think task and the intrusions rating scale. This practice was run in three stages and the two filler titles were used as memory cues in this phase.

In stage one, participants practiced thinking and not-thinking about associated memories without the intrusions rating scale. This stage consisted of 10 trials. The two filler items were presented randomly and evenly across all trials. In the second stage, participants practiced responding to a rating scale: One of three scale labels were presented on the screen for 4000ms, followed by the rating scale (1 2 3). Participants responded to the scale using the 1, 2, 3 number keys on the keyboard, and the next trial began only if the response was accurate. Each label was randomly repeated thrice, leading to a total of nine trials in this stage. In the third stage, participants practiced a combination of the Think/No-Think task and the rating scale, mirroring the experimental Think/No-Think phase. The two filler items were assigned to the same condition as in stage one, but the order of presentation of Think/No-Think cues was randomised again. This stage consisted of 10 trials. Following the third stage, the main experiment began.

After both stages one and three, the experimenter orally administered a diagnostic questionnaire to ensure that the participants were accurately following the instructions. The experimenter then gave feedback to the participant after each question to reinforce the instructions. This diagnostic questionnaire was also administered at the halfway point of the experimental Think/No-Think phase.

**Surprise memory test.** After the Think/No-Think task on Day 2, participants completed a surprise test of the autobiographical memories associated with all cues during the final phase of the experiment. See Figure S.1. for an illustration. They were instructed to disregard all previous tasks and recall the memory for each cue regardless of the colour it had been displayed in during the previous phase. Like in session one, participants typed descriptions of the memories in a text box on the computer using a keyboard. In this phase, a cue was displayed on the screen for 10s and participants pressed a button as soon as the associated memory came to mind, which was followed by a text box. They had two minutes to describe the associated memory in as much detail as possible. They then reported same memory characteristics as in session one: memory age, vividness, intentionality, morality rating, I-PANAS-SF, and SAM measures. See Figure S.1. for an illustration of the whole experimental procedure.

**Analysis strategy to measure suppression-induced changes in autobiographical memory descriptions**

Each memory description that participants provided was manually coded on the following measures: i) Specificity, which was quantified by identifying four core aspects of the memory event – *who* was involved, *what* happened, *where* did the event take place, and *when* did it take place. As these were moral memories of personal actions towards others, the “who” and “what” aspects were prioritised. This specificity measure was rated on a Likert scale ranging from *1 = Not at all specific* to *5 = Extremely specific*; ii) Emotional valence was quantified by identifying specific positive or negative words that conveyed the emotion felt by the participant. This was rated on a scale of *1 = Very negative, 4 = Not negative or positive, 7 = Very positive*. Finally, for each memory, the iii) similarity of both descriptions were rated on a general intuitive sense of similarity between the descriptions. Importantly, this measure was used to test if the content of the memory was changed or forgotten between the first and second report. A 5-point scale was used to measure similarity, *1 = Not at all similar, 5 = Extremely similar*.

The experimenter rated all memories in the study and five different independent coders second rated 50% of the memories (10% each coder). Although all coders had prior knowledge of the experimental design, they were blind to whether the memories had been assigned to Think or No-Think instructions and did not know if the descriptions were from before or after the Think/No-Think task. Due to the nature of the memory descriptions, it was not possible to blind the coders to whether the memories were morally wrong or right. The correlation coefficient between the experimenter and independent coders was .75 (ranging from .66 to .89), suggesting good agreeability between coders. Only the experimenter’s ratings were used for further analyses since only the experimenter rated all memory description.


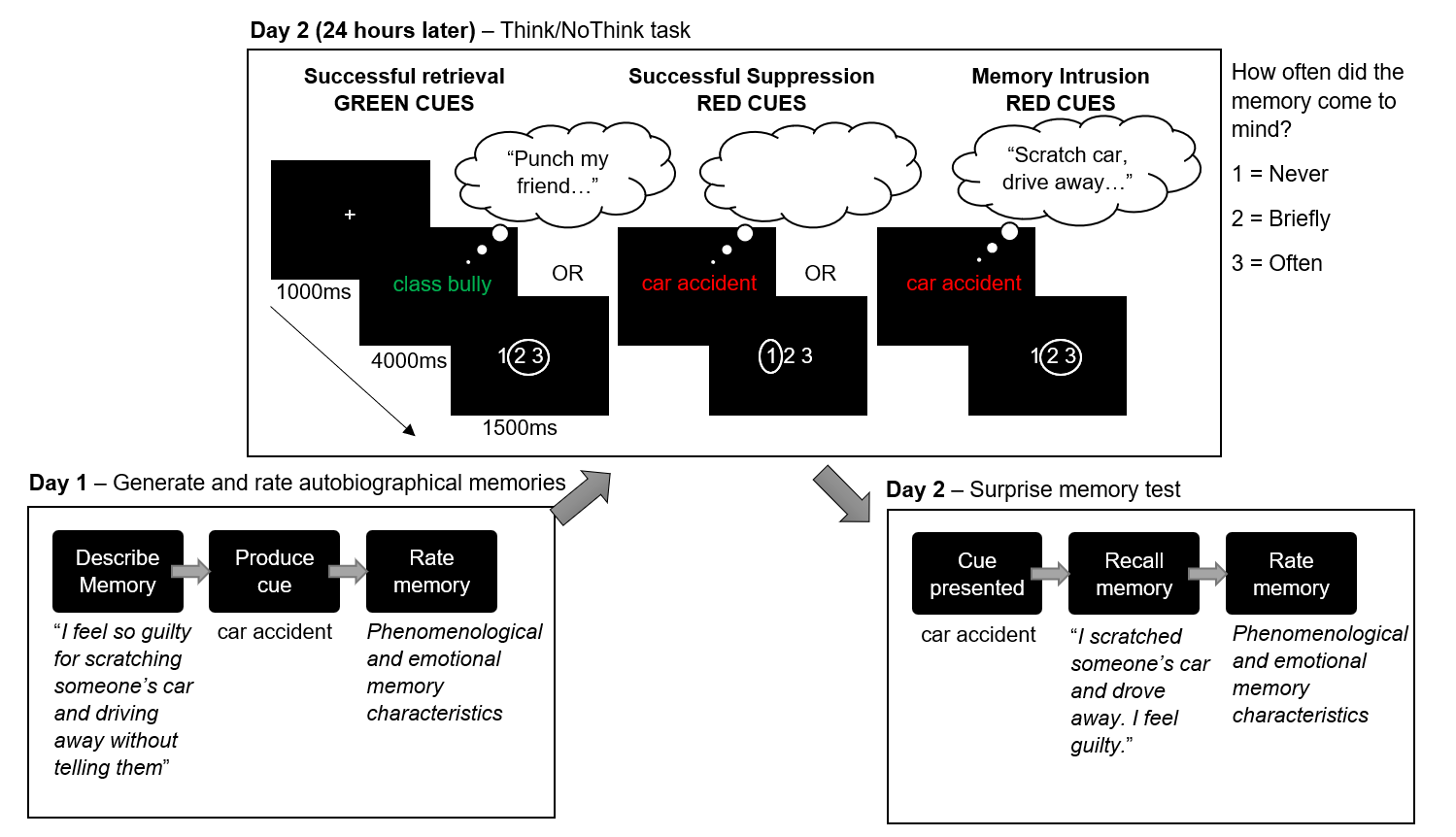
Figure S.1. Illustration of one trial in the critical procedural phases in the experiment. Participants attempted to suppress (No-Think) memories associated to red cues, but consciously recollect the associated memories green cues. Participants indicated how often the associated memory came to mind using the “1 2 3” rating scale.

**Supplementary Results**

**Phenomenological and emotional characteristics of autobiographical memories**

A difference score was computed between ratings reported in the first session and the second session, and this was used as a dependent variable to test the effect of the TNT manipulation on phenomenological characteristics of memories (see Figure S.2).


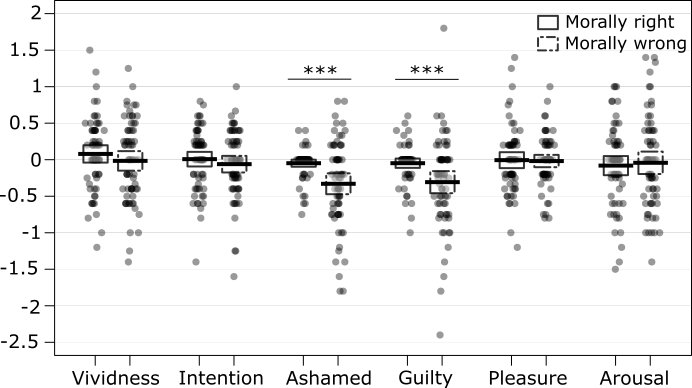


*Figure S.2.* Average of difference score (before TNT – after TNT) compared across memory type. The scatter dots show difference scores of each individual for each. The thick lines show the group means and the boxes depict the 95% confidence interval of the group means.****p <.001.*

A 2 (Instruction Type: Think, No-Think) x 2 (Memory Type: Morally right, morally wrong) repeated measures ANOVA was conducted to test the effect of the TNT task on the difference score of the chosen self-report measures. See Table S.1. for the results from the ANOVAs.

Neither the main effects of instruction type, nor instruction type x memory type interactions were significant for any measure. However, there were significant main effects of memory type for some measures: Participants reported feeling less ashamed and guilty for morally wrong memories after the TNT task, but there were no such changes in guilt and shame for morally right memories.

Table S.1. ANOVA results from omnibus test with the difference score (After TNT – Before TNT).

|  | *Phenomenological memory characteristics* | | | | | | | | | | | | |
| --- | --- | --- | --- | --- | --- | --- | --- | --- | --- | --- | --- | --- | --- |
|  | Memory Age | | | Vividness | | | Intention | | | Morality | | | |
|  | F | p | *η*^2^_p_ | F | p | *η*^2^_p_ | F | p | *η*^2^_p_ | F | p | *η*^2^_p_ |  |
| IT (T vs. NT) | 0.01 | 0.91 | 0.00 | 0.00 | 0.95 | 0.00 | 0.05 | 0.82 | 0.00 | 0.47 | 0.50 | 0.01 |  |
| MT (MW vs. MR) | 0.44 | 0.51 | 0.01 | 2.07 | 0.16 | 0.06 | 0.51 | 0.48 | 0.02 | 0.01 | 0.91 | 0.00 |  |
| ITxMT | 0.61 | 0.44 | 0.02 | 0.68 | 0.42 | 0.02 | 0.49 | 0.49 | 0.02 | 1.51 | 0.23 | 0.04 |  |
|  | *I-PANAS-SF* | | | | | | *SAM* | | | | | |  |
|  | Ashamed | | | Guilty | | | Pleasure | | | Arousal | | | |
|  | F | p | *η*^2^_p_ | F | p | *η*^2^_p_ | F | p | *η*^2^_p_ | F | p | *η*^2^_p_ |  |
| IT (T vs. NT) | 0.31 | 0.58 | 0.01 | 0.19 | 0.66 | 0.01 | 0.15 | 0.70 | 0.01 | 2.35 | 0.14 | 0.07 |  |
| MT (MW vs. MR) | 12.42 | 0.001 | 0.27 | 8.93 | 0.005 | 0.21 | 0.03 | 0.88 | 0.00 | 0.30 | 0.59 | 0.01 |  |
| ITxMT | 0.69 | 0.41 | 0.02 | 2.23 | 0.14 | 0.06 | 0.00 | 0.96 | 0.00 | 0.36 | 0.55 | 0.01 |  |

Note. IT = Instruction Type, MT = Memory Type, T = Think, NT = No-Think, MW = Morally Wrong, MR = Morally Right. *η*^2^_p_ = partial eta sq. (effect size). I-PANAS-SF = International – Positive and Negative Affect Scale – Short Form, SAM = Self-Assesssment Manikin. I-PANAS-SF = International – Positive and Negative Affect Scale – Short Form. Significant effects (*p <* .05) are shown in bold. *N* *= 34*.

**Results for suppression-induced changes in autobiographical memory descriptions**

A 2 (Instruction Type) x 2 (Memory Type) repeated measures ANOVA was first conducted on the ratings of how similar the memory descriptions were that participants provided before and after the Think/No-Think task (see Table S.2).

Table S.2. Average similarity ratings across instruction and memory types. Ratings were provided by the experimenter

|  | Experimenter ratings | | Independent ratings | |
| --- | --- | --- | --- | --- |
|  | Right | Wrong | Right | Wrong |
| Think | 4.07(.43) | 3.84(.54) | 3.83(.93) | 3.77(.97) |
| No-Think | 4.05(.52) | 3.65(.51) | 3.71(1.09) | 3.80(.96) |

Note. Similarity was rated on a 5-point scale (1 = Not at all similar, 5 = Extremely similar). Standard deviation is reported in brackets.

Although memory descriptions in the No-Think condition were rated as overall less similar than descriptions in the Think condition, this difference was not significant¸ *F*(1,33) = 2.17, *p* = .15, partial *η*^2^ = .065. There was however a significant main effect of memory type, as morally wrong memories (*M* =3.75, *SD* = .48) were rated as overall less similar across the two times than morally right memories (*M* = 4.06, *SD* = .37); *F*(1,33) = 18.27, *p* < .001, partial *η*^2^ = .356. The interaction between instruction type and memory type was not significant; *F*(1,33) = 2.14, *p* = .15, partial *η*^2­^ = .06.

Next, a 2 (Instruction Type) x 2 (Memory Type) x 2 (Administration time: Before TNT vs After TNT) repeated-measures ANOVA was conducted on the rating of specificity and valence of the memory descriptions (Table S.3.).

Table S.3. Average ratings of specificity and valence across instruction and memory types. Ratings were provided by the experimenter.

|  | Experimenter ratings | | | | Independent ratings | | | |
| --- | --- | --- | --- | --- | --- | --- | --- | --- |
|  | Specificity | | | | | | | |
|  | Right | | Wrong | | Right | | Wrong | |
|  | Pre-TNT | Post-TNT | Pre-TNT | Post-TNT | Pre-TNT | Post-TNT | Pre-TNT | Post-TNT |
| Think | 2.93(.64) | 2.93(.67) | 2.95(.63) | 2.94(.65) | 2.95(.99) | 3.10(1.06) | 2.91(.63) | 3.08(.65) |
| No-Think | 2.96(.59) | 2.91(.69) | 2.93(.72) | 2.95(.68) | 3.14(1.00) | 3.00(1.10) | 3.09(1.06) | 3.16(1.04) |
|  | Valence | | | | | | | |
| Think | 4.21(.35) | 4.20(.46) | 3.35(.51) | 3.84(.46) | 4.45(.88) | 4.48(.84) | 3.16(.81) | 3.19(.84) |
| No-Think | 4.15(.43) | 4.26(.48) | 3.35(.59) | 3.35(.57) | 4.49(.97) | 4.22(.90) | 3.30(1.06) | 3.28(.88) |

Note. Specificity was rated on a 5-point scale (1 = Not at all specific to 5 = Extremely specific), emotional valence was measured on a 7-point scale (1 = Very Negative, 4 = Not negative or positive, 7 = Very Positive). Standard deviation is reported in brackets.

Unsurprisingly, descriptions of morally right memories were rated as containing more positively valanced words than morally wrong memories *F*(1,33) = 42.01, *p* < .001, partial *η*^2^ = .56. For both measures, neither the main effects of administration time, nor its interaction with instruction type and memory type were significant (all *Fs* < 2.34, *ps* > .14).

Thus, both self-reports and experimenter’s ratings showed that morally wrong memories changed between the first and second session in terms of how they were experienced and described by participants, but no such changes occurred for the morally right memories, and the changes were not influenced by the Think/No-Think manipulation.
